# Genetic insight into putative causes of xanthelasma palpebrarum: a Mendelian randomization study

**DOI:** 10.3389/fimmu.2024.1347112

**Published:** 2024-03-27

**Authors:** Wenting Hu, Yaozhong Liu, Cuihong Lian, Haocheng Lu

**Affiliations:** ^1^ Department of Dermatology, Shenzhen Second People’s Hospital, Shenzhen, Guangdong, China; ^2^ Department of Internal Medicine, Cardiovascular Center, University of Michigan Medical Center, Ann Arbor, MI, United States; ^3^ Department of Pharmacology, Joint Laboratory of Guangdong-Hong Kong Universities for Vascular Homeostasis and Diseases, School of Medicine, Southern University of Science and Technology, Shenzhen, Guangdong, China

**Keywords:** Mendelian randomization, Xanthelasma palpebrarum, plasma lipid, circulating protein, cytokine

## Abstract

Xanthelasma palpebrarum (XP) is the most common form of cutaneous xanthoma, with a prevalence of 1.1%~4.4% in the population. However, the cause of XP remains largely unknown. In the present study, we used Mendelian randomization to assess the genetic association between plasma lipids, metabolic traits, and circulating protein with XP, leveraging summary statistics from large genome-wide association studies (GWAS). Genetically predicted plasma cholesterol and LDL-C, but not HDL-C or triglyceride, were significantly associated with XP. Metabolic traits, including BMI, fasting glucose, type 2 diabetes, systolic and diastolic blood pressure, were not significantly associated with XP. Furthermore, we found genetically predicted 12 circulating proteins were associated with XP, including FN1, NTM, FCN2, GOLM1, ICAM5, PDE5A, C5, CLEC11A, CXCL1, CCL2, CCL11, CCL13. In conclusion, this study identified plasma cholesterol, LDL-C, and 12 circulating proteins to be putative causal factors for XP, highlighting the role of plasma cholesterol and inflammatory response in XP development.

## 1 Introduction

Xanthelasma palpebrarum (XP) is the most common form of cutaneous xanthoma, usually manifesting as bilateral, symmetrical, soft, yellowish papules and plaques over the eyelid (1). A large cohort in Denmark shows that the prevalence of XP in the general population is about 4.4% (2). The prevalence varies in other studies (1.1%~4.4%) (3), possibly due to its usually asymptomatic nature, and many patients do not get diagnosed. Patients often seek medical help because of its significant cosmetic burden and request treatment for these aesthetically undesirable facial lesions (4). Several therapeutic methods have been developed for XP, including surgical excision, laser therapy, chemical peeling, cryotherapy, radiofrequency ablation, plasma sublimation, and dermabrasion (5). Although surgical excision is the most common method, it could result in complications, such as scarring, dyspigmentation, and ectropion (5). Recurrence of XP is common, regardless of treatment method, ranging from 40% to 80% (6, 7). Currently, a gold-standard long-term treatment option has yet to be established (8), attributed to our limited understanding of the pathogenesis of XP.

XP lesions comprise mainly foamy histiocytes located within the upper reticular dermis or in perivascular and periadnexal areas, and the intracellular vacuoles contain esterified cholesterol and lipids, similar to evolving atheromas (4, 9). XP is a common complication in patients with familial hypercholesterolemia (10, 11). Many studies demonstrated that XP is associated with dyslipidemia, particularly high total cholesterol (TC) and high low-density lipoproteins (LDL) (12–14). From a meta-analysis of over 854 XP patients, XP patients had significantly higher serum levels of total cholesterol and LDL, higher apolipoprotein B, and relatively lower apolipoprotein A1. No significant difference in high-density lipoprotein (HDL), very low-density lipoprotein (VLDL), and triglyceride (TG) was observed between XP patients and the control population. Notably, about half of XP patients show normal lipid profiles (15). Nevertheless, it is still controversial whether hyperlipidemia is a cause of XP or just associated with XP because of possible potential confounding factors and reverse causation bias.

Mendelian randomization (MR) uses genetic variations to address causal relationships between modifiable or unmodifiable exposure and outcomes (16). MR is based on instrumental variable (IV) analysis. A validated IV is strongly related to exposure but not to the outcome, except through its association with exposure. The careful selection of IV could infer the causality between exposure and outcome in the presence of unobserved confounding factors. Here, we applied MR analysis to address the genetic causal effects of plasma lipids, metabolic traits, and circulating proteins on XP (17, 18) to investigate the molecular mechanisms and find potential drug targets for XP.

## 2 Method

### 2.1 Sources of exposure and outcome datasets

The study relied on publicly available summary statistics from large-scale GWAS. The datasets used in this study are listed in **Supplementary Table 1**. The FinnGen combines the imputed genotype and digital health record data from the Finnish population. It is the largest GWAS dataset available containing the XP phenotype with 228 cases and 344684 controls.

### 2.2 Selection of instrumental variables

We selected genome-wide significant single nucleotide polymorphisms (SNP) whose p-value is less than 5*10^-8^ for the plasmid lipid and metabolic traits. We excluded correlated SNPs whose linkage disequilibrium (LD) R2 > 0.001 in the 10,000kb region. We only keep the SNPs with F statistics > 10 to avoid weak IV bias. To prevent reverse correlation, we only included SNPs that explain a substantially larger variance of exposures than outcomes as calculated by the Steiger filter test (p< 0.05). IVs associated with possible confounding factors are removed by using PhenoScanner V2 tool (19).

For the circulating proteins, we used consortium of deCODE genetics (20), which contains the association between genetic variants and 4719 plasma proteins in 35,559 Icelanders (21). Valid SNPs were selected based on the following criteria: p-value< 5*10^-8^; Steiger filtering test p<0.05; cis-pQTL, which SNPs within 1MB from gene starting site; clumped to conditionally independent genetic IVs (R^2^< 0.001 and kb=10,000). For proteins with more than 1 IV, the inverse variance-weighted (IVW) method was used; For proteins with only 1 IV, the Wald ratio method was used.

For the plasma inflammatory proteins, we used (1) GWAS results from The Cardiovascular Risk in Young Finns Study (YFS), which is a multicenter follow-up study with randomly chosen subjects from the Finnish cities of Helsinki, Kuopio, Oulu, Tampere, and Turku and their rural surroundings (22, 23). In this study, a total of 41 cytokines in the plasma were measured; (2) GWAS summary statistics from SCALLOP Consortium meta-analysis GWAS summary statistics for the Olink Inflammation panel (24). In this study, a genome-wide protein pQTL of 91 plasma proteins was measured using Olink Target platform in 14,824 participants. For both studies, valid SNPs were selected based on the following criteria: p-value< 5*10^-8^; Steiger filtering test p<0.05; both cis- and trans- SNPs were used; clumped to conditionally independent genetic IVs (R^2^< 0.001 and kb=10,000). For proteins with more than 1 IV, the inverse variance-weighted (IVW) method was used; For proteins with only 1 IV, the Wald ratio method was used.

### 2.3 Two-sample Mendelian randomization

The two-sample MR was performed using TwoSampleMR (25) and MendelianRandomization (26) R package. For each exposure, we retrieved the summary statistics of selected IVs from the outcome dataset (27) to perform MR analysis. Data on exposure and outcome were then harmonized to ensure that the effect of an SNP on exposure and outcome corresponded with the same allele. *F* statistics were calculated by sample size (*N*) and number of instruments (*K*) as: $F=(\frac{N-K-1}{K})(\frac{R^{2}}{1-R^{2}})$ *R* ^2^ is the variance in the exposure explained by the genetic variant, calculated as $R^{2}=2\beta_{X}^{2}MAF(1-MAF)$ (*MAF* is the minor allele frequency). To avoid the weak IV bias, SNPs with *F*< 10 were excluded in the MR analysis. In the primary analysis, we used the inverse variance-weighted (IVW) method, which provided the highest precision, assuming that all IVs are valid (28). The Wald ratio estimate of the *j*th variant is: ${\hat{\theta }}_{j}=\frac{{\hat{\beta }}_{Yj}}{{\hat{\beta }}_{Xj}}$ and its approximate standard error is $se({\hat{\theta }}_{j})=\left|\frac{se({\hat{\beta }}_{Yj})}{{\hat{\beta }}_{Xj}}\right|$

The IVW estimate can be expressed as:


$${\hat{\theta }}_{\text{IVW}}=\frac{\sum_{j}{\hat{\theta }}_{j}\mathrm{se}{\left({\hat{\theta }}_{j}\right)}^{-2}}{\sum_{j}\mathrm{se}{\left({\hat{\theta }}_{j}\right)}^{-2}}=\frac{\sum_{j}{\hat{\beta }}_{Yj}{\hat{\beta }}_{Xj}\mathrm{se}{\left({\hat{\beta }}_{Yj}\right)}^{-2}}{\sum_{j}{{\hat{\beta }}_{\mathrm{Xj}}}^{2}\mathrm{se}{\left({\hat{\beta }}_{Yj}\right)}^{-2}}$$

The standard error of the IVW estimate is:


$$\mathrm{se}({\hat{\theta }}_{\text{IVW}})=\sqrt{\frac{1}{\sum_{j}{{\hat{\beta }}_{\mathrm{Xj}}}^{2}\mathrm{se}{\left({\hat{\beta }}_{Yj}\right)}^{-2}}}$$

MR analyses were conducted using R (version 4.3.2) package “TwoSampleMR v0.5.8”. The weighted median method is used, assuming that at least half of the IVs are valid. In addition, the MR-Egger method was used to correct the potential horizontal pleiotropy. The Cochran Q heterogeneity test (using TwoSampleMR::mr_heterogeneity function) was used to determine heterogeneity. The MR-Egger intercept test (using TwoSampleMR::mr_pleiotropy_test function)was used to determine the unbalanced pleiotropy between exposure and outcome. The Steiger directionality test (using TwoSampleMR::directionality_test function) was used to determine the causal direction of the exposure and outcome. Sensitivity analysis was conducted by leave-one-out analysis (using TwoSampleMR:: mr_leaveoneout function). For circulating proteins and cytokines, the p-value was corrected by the FDR method.

## 3 Results

### 3.1 The causal effect of plasma lipids on XP

**Figure 1:**
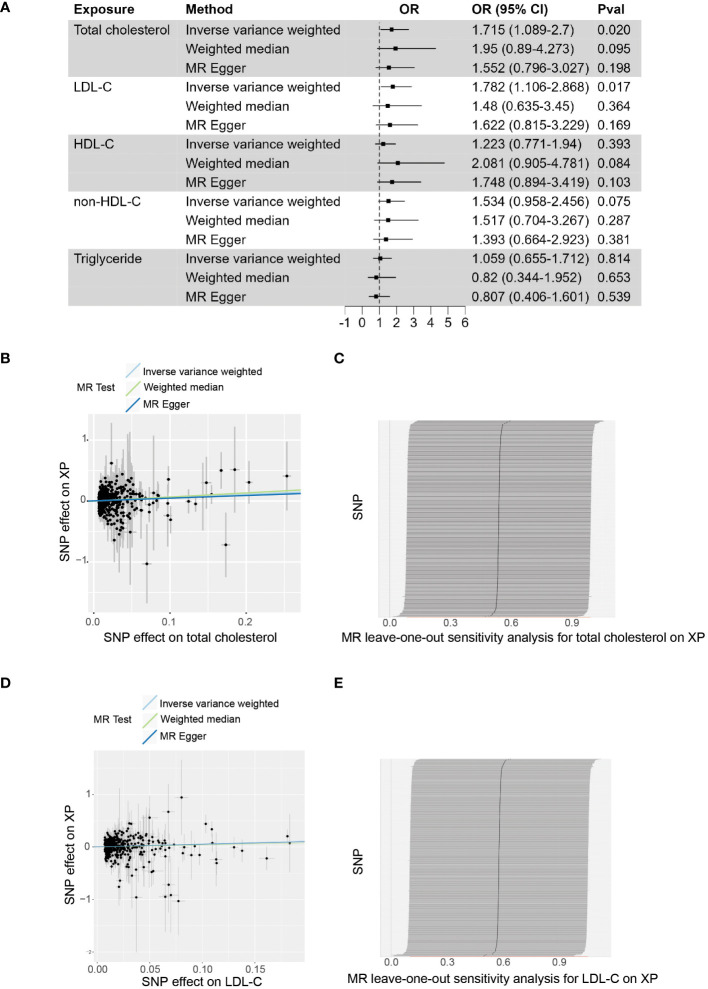
The causal effect of plasma lipids on XP. **(A)**, Forest plot to visualize the causal effect of plasma lipids on XP. **(B)**, Scatter plot to visualize the causal effect of plasma total cholesterol on XP. **(C)**, Leave-one-out plot to visualize the causal effect of total cholesterol on XP when leaving one SNP out. **(D)**, Scatter plot to visualize the causal effect of plasma LDL-C on XP. **(E)** Leave-one-out plot to visualize the causal effect of LDL-C on XP when leaving one SNP out. OR, Odds ratio; CI, confidence interval; Pval, p-value; LDL-C, low-density lipoprotein cholesterol; HDL-C, high-density lipoprotein cholesterol; non-HDL-C, non-high-density lipoprotein cholesterol.

Based on the up-to-date largest GWAS study of XP from FinnGen Datafreeze 9 release and lipid traits from Global Lipids Genetics Consortium (2021), we assessed the causal effects of lipid traits and XP diseases, using two sample MR methods (**Figure 1A**; **Supplementary Table 2**). In the primary inverse variance weighted (IVW) analysis, we found that genetically predicted total cholesterol (Odds ratio, OR, 1.715; 95% confidence intercept, CI, 1.089 - 2.7; p = 0.020) and LDL (OR, 1.782; CI, 1.106-2.868; p = 0.017) were significantly associated with XP (**Figures 1B, D**). The leave-one-out analysis showed the robustness of the MR estimates (**Figures 1C, E**). The alternative analysis with the Weighted median and MR Egger method shows the same direction but did not reach significance, likely because they are of less power. For other lipid traits, including HDL (OR, 1.223; CI, 0.771-1.94; p = 0.393), non-HDL cholesterol (OR, 1.534; CI, 0.958-2.456; p = 0.075), and triglyceride (OR, 1.059; CI, 0.655-1.712; p = 0.814, the association did not reach statistical significance in primary IVW analysis.

### 3.2 The causal effect of metabolic traits on XP

**Figure 2:**
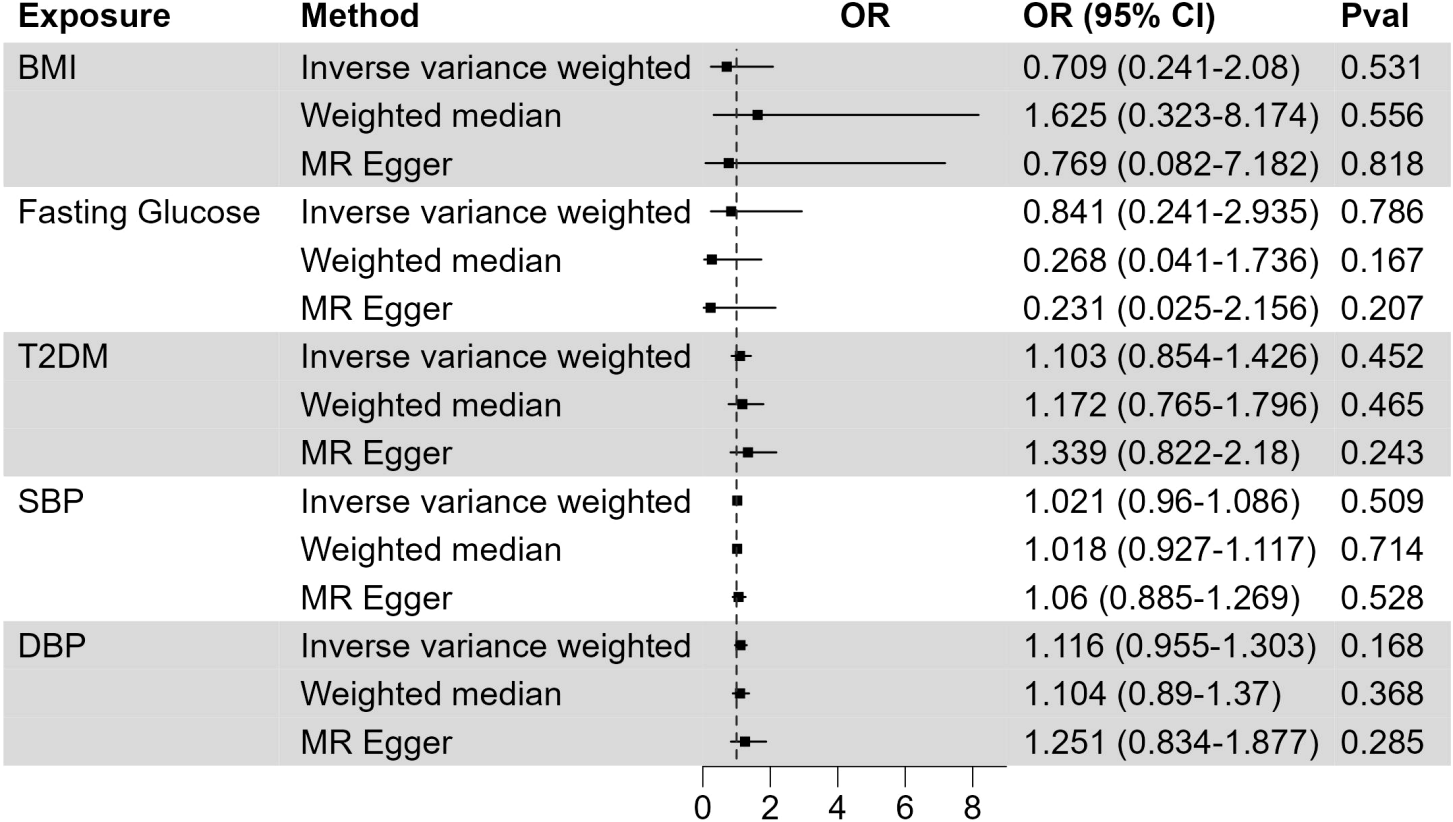
The causal effect of metabolic traits on XP. Forest plot to visualize the causal effect of BMI, fasting glucose, type 2 diabetes, systolic blood pressure, and diastolic blood pressure on XP. OR, Odds ratio; CI, confidence interval; Pval, p-value; BMI, body mass index; T2DM, Type 2 Diabetes Mellitus; SBP, systolic blood pressure; DBP, diastolic blood pressure.

XP was reported to be associated with other metabolic traits, including body weight, blood glucose, and blood pressure (29). We used large GWAS studies of BMI, diabetes, and blood pressure to assess their possible causality to XP (**Figure 2**; **Supplementary Table 3**). In primary IVW analysis, genetically predisposition of increased BMI (OR, 0.709; CI, 0.241-2.08; p = 0.531), fasting glucose (OR, 0.841; CI, 0.241-2.935; p = 0.786), type 2 diabetes mellitus (T2DM) (OR, 1.103; CI, 0.854-1.426; p = 0.452), systolic blood pressure (OR, 1.021; CI, 0.96-1.086; p = 0.509), diastolic blood pressure (OR, 1.116; CI, 0.955-1.303; p = 0.168) did not significantly increase the risk of XP. These metabolic traits did not significantly increase the risk of XP in alternative Weighted median and MR Egger methods.

### 3.3 Putative causal circulating proteins on XP

**Figure 3:**
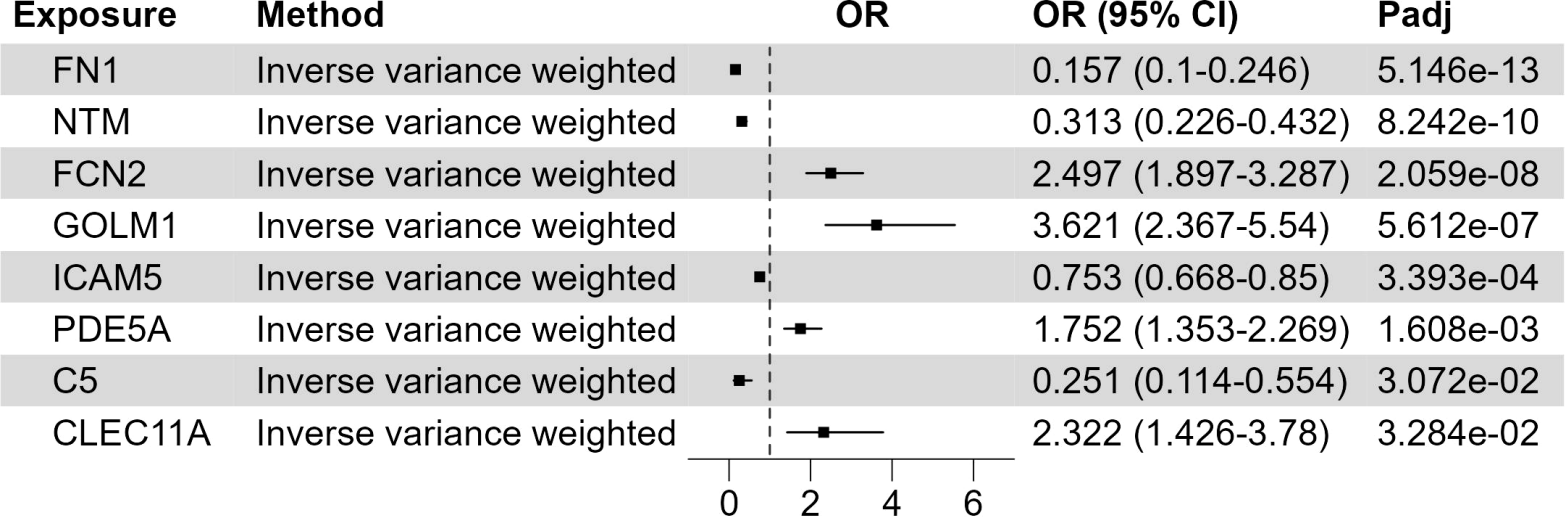
Putative causal circulating proteins on XP. Forest plot to visualize the causal effect of circulating proteins on XP. OR, Odds ratio; CI, confidence interval; Padj, FDR corrected p value. Candidates are selected based on the following criteria: MR results p-value (FDR adjusted)< 0.05; Egger Intercept P value > 0.05; Correct causal direction; Steiger P-value< 0.05; Q p value > 0.05.

The development of XP involves the trans-endothelial migration of immune cells and their uptake of lipids. To identify the possible circulating proteins regulating these processes, we utilize the large-scale GWAS of circulating proteins (20). The protein quantitative trait loci (pQTLs) are used as genetic IVs in the MR study, and we integrate the plasma proteome with the XP GWAS (**Figure 3**; **Supplementary Table 4**). We found at least 1 SNPs as validated IV for 1199 circulating proteins and used these SNPs as genetic predictors of protein expression. Two sample MR analysis found 8 proteins with FDR corrected p-value< 0.05: fibronectin 1 (FN1, p = 5.15*10^-13^), neurotrimin (NTM, p = 8.24*10^-10^), ficolin 2 (FCN2, p = 2.06*10^-8^), Golgi membrane protein 1 (GOLM1, p = 5.61*10^-7^), intercellular adhesion molecule-5 (ICAM5, p = 3.9*10^-4^), phosphodiesterase 5A (PDE5A, p = 1.6*10^-3^), Complement C5 (C5, p = 0.031), C-type lectin domain containing 11A (CLEC11A, p = 0.033). Among them, FN1, NTM, ICAM5, and C5 were negatively associated with XP, while FCN2, GOLM1, PDE5A, and CLEC11A were positively associated with XP.

### 3.4 Putative causal circulating inflammatory proteins on XP

**Figure 4:**
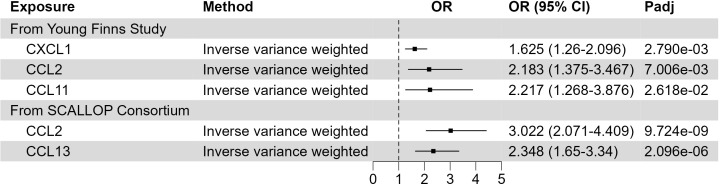
Putative causal inflammatory proteins on XP. Forest plot to visualize the causal effect of inflammatory proteins on XP. OR, Odds ratio; CI, confidence interval; Padj, FDR corrected p value. Candidates are selected based on the following criteria: MR results p-value (FDR adjusted)< 0.05; Egger Intercept P value > 0.05; Correct causal direction; Steiger P-value< 0.05; Q p value > 0.05.

Inflammation plays an important role in various skin diseases, such as psoriasis and eczema. However, the role of inflammatory response in XP is still not clear. To address this question, we leveraged the GWAS data of inflammatory proteins (22–24). Using two-sample MR, we found that 3 out of 41 cytokines in Young Finns Study (**Figure 4**; **Supplementary Table 5**) were significantly associated with XP (FDR corrected p-value< 0.05), including C-X-C motif chemokine ligand 1 (CXCL1, also named GRO alpha, p = 0.0028), C-C motif chemokine ligand 2 (CCL2, also called MCP1, p = 0.0070), and C-C motif chemokine ligand 11 (CCL11, also name eotaxin, p = 0.026). In SCALLOP Consortium GWAS results, we found 2 out of 91 proinflammatory proteins were significantly associated with XP, including CCL2 (p = 9.724*10^-9^) and CCL13 (2.096*10^-6^) (**Supplementary Table 6**). All the cytokines were positively associated with XP, indicating a possible role of inflammation in XP.

## 4 Discussion

XP is a relatively common skin disease, but the actual cause is still not clear. In this study, we used a two-sample MR analysis to investigate the risk factors and mechanism of XP. We used large lipid GWAS data and found that total cholesterol and LDL-C were causally associated with XP. In addition, to further understand the molecular mechanism of XP, in GWAS data of circulating protein and cytokines, we identified 8 circulating proteins and 3 inflammatory cytokines to be putative causes and potential therapeutic targets of XP in patients.

Many observation studies have reported that XP patients have significantly higher levels of cholesterol and LDL-C but various levels of triglycerides and HDL-C. Most studies only have tens or hundreds of individuals in the cohort, which limits the power of statistical analysis. XP is pathologically characterized by infiltration of lipid-rich foam cells in the demis, which shares many similarities with atherosclerosis. Given that high cholesterol, especially LDL-C is well established as a risk factor for atherosclerosis, XP is also often attributed to high plasma cholesterol. However, the causal effects of plasma lipids and XP is still not clear. The association of XP and high cholesterol could be attributed to other confounding factors, such as diet or lifestyle, which are difficult to exclude in observation studies. To address these issues, we conducted a comprehensive MR analysis to determine the causality between plasma lipids and XP. The MR analysis used genetic variants as IV to test the causality and could overcome the problem of confounding factors and reverse causality. All the SNPs used in the study were listed in (**Supplementary Table 7**). We used the FinnGen Datafreeze 9 release (27) as the GWAS dataset for XP and the Global Lipids Genetics Consortium Results (GLGC) (30) as GWAS for plasma lipids. GLGC is a multi-ancestry meta-analysis of lipid levels in more than 1.65 million individuals. Two-sample MR analysis performed on these 2 datasets demonstrated that genetically predicted higher plasma total cholesterol and LDL-c, but neither triglycerides nor HDL-C were causally associated with XP. The horizontal pleiotropy was checked by Egger intercept, and heterogeneity was checked by Cochran’s Q test. The alternative Weighted median and MR Egger test showed consistent direction but no significance, possibly due to the low accuracy of these methods compared with the primary IVW method. This analysis provides direct evidence that high plasma cholesterol and high LDL-C are both causal factors of XP, and cholesterol or LDL-C lowering therapy could be helpful in XP patients.

Many studies have shown that higher plasma lipids increase XP risk, but about half of XP patients exhibit a normal plasma lipid profile. This phenomenon indicates that there are additional pathways that determine XP development. In atherosclerosis development, the lesion is initiated by the trans-endothelial migration of monocytes into the aortic wall, followed by the uptake of lipids by monocyte-derived macrophage in the lesion (31). The infiltration of monocytes to tissue is regulated by many proinflammatory cytokines and chemotactic factors (32). To assess whether these processes contribute to lipid accumulation in the XP lesion, we used the pQTL data from a large GWAS dataset of circulating proteins and inflammatory cytokines to perform the two-sample MR analysis on XP. The analysis of circulating proteins showed that 8 proteins in the plasma could causally associate with XP, including FN1, NTM, FCN2, GOLM1, ICAM5, PDE5, C5, and CLEC11A (**Figure 3**).

Fibronectin (FN1) is a glycoprotein with multiple variants. FN1 is an extracellular matrix protein and plays an important role in cell adhesion, migration, growth, and differentiation (33, 34). Neurotrimin (NTM) is a neural cell adhesion molecule that regulates neurite outgrowth (35). Intriguingly, NTM is also implicated in several cardiovascular diseases, including heart failure (36), hypertension (37) and aneurysm (38). Ficolin2 (FCN2) is a soluble collagen-like protein that binds to pathogen pattern molecules (39) and is involved in innate immune defense (40). Golgi Membrane Protein 1 (GOLM1) is mainly located in the Golgi membrane and can also be secreted into circulation (41). Circulating GOLM1 can be used as an early diagnosis marker of hepatocellular carcinoma (42, 43). GOLM1 is also implicated in other cancers, including melanoma (44) and colon cancer (45). Intercellular adhesion molecule 5 (ICAM5) is an adhesion molecule important for the recruitment of inflammatory cells to the sites of inflammation. ICAM5 is also important for pathogen infection (46), auto-immune disease (47, 48), and nervous system (49, 50). Phosphodiesterase type 5A (PDE5A) selectively hydrolyzes cyclic GMP and is critical for maintaining cardiovascular homeostasis (51). PDE5 regulates vascular tone through the NO-cGMP pathway (52), and PDE5 inhibitors show promising results in treating heart ischemia injury (53, 54) and pathological hypertrophy (55). Complement factor C5 (C5) is a key component of the complement system and innate immune system. C5 is cleaved to C5a and C5b by C5 convertase. C5a functions as a potent chemotactic factor, and C5b facilitates the assembly of membrane attack complex (56). C5 inhibitors have been approved for the treatment of diseases of complement overactivation, including paroxysmal nocturnal hemoglobinuria, atypical hemolytic uremic syndrome, and vasculitis (57). C-type lectin domain family 11, member A (CLEC11A) is an osteogenic growth factor and is important for maintaining an adult skeleton (58, 59).

In the development of atherosclerosis, the recruitment of immune cells to the vascular wall is induced by several inflammatory cytokines and chemotactic factors. To address whether this process was also implicated in the accumulation of lipid-laden cells in the XP lesion, we utilized the GWAS dataset of 41 inflammatory cytokines in the blood (22, 23). In this dataset, we identified 3 cytokines, including CXCL1, CCL2, and CCL11, that were positively associated with XP. CXCL1 and its receptor CXCR2 signaling are crucial for monocyte infiltration into inflammatory tissues. CXCL1/CXCR2 signaling are widely studied in cardiovascular diseases, including cardiac hypertrophy (60), hypertension (61), aneurysm (62), and atherosclerosis (63). CCL11 [eosinophil chemotactic protein (Eotaxin)] has a selective role in the recruitment of eosinophils via activating CCR2, CCR3, and CCR5 receptors (64). Increased circulating CCL11 was implicated in several auto-immune and allergic diseases, including systemic lupus erythematosus (65), asthma (66), and multiple sclerosis (67). Importantly, the association between circulating CCL2 and XP was cross-validated in 2 different GWAS results. CCL2, also known as monocyte chemoattractant protein-1 (MCP-1), is a key chemoattractant protein for monocytes. By binding to its primary receptor, CCR2, CCL2 coordinates inflammatory monocytes traveling among bone marrow, blood, and inflammatory tissue (68). CCL2 can also regulate the migration and infiltration of other immune cells, including memory T lymphocytes and natural killer (NK) cells (69). CCL13 (MCP4) could induce the chemotaxis of multiple immune cells, including eosinophils, basophils, monocytes, macrophages, immature dendritic cells, and T cells (70). CCL13 is implicated in asthma, rheumatic diseases, skin conditions (atopic dermatitis and alopecia areata), and cancer (71). All of these cytokines are positively associated with XP and could potentially serve as novel therapeutic targets in XP.

Many studies reported the association between high plasma cholesterol and XP, but the mechanism of accumulation of lipids in the soft tissue is still not clear. XP is pathologically similar to atherosclerosis, characterized by infiltration of lipid-rich foam cells and proliferation of endothelial and fibroblastic cells (5). In the development of atherosclerosis, the infiltration of macrophages, followed by lipid uptake by infiltrated cells, is a key step in the progression of the disease. In this study, we found several plasma proteins involved in the inflammatory process (FCN2, C5, CXCL1, CCL2, CCL11, and CCL13) and cell adhesion (NTM and ICAM5) to be associated with XP. This information supports that local inflammation could be involved in the XP and deserves further investigation.

Our study has certain strengths. First, unlike observation studies, MR analysis could test the causality between exposures and outcomes, while observational studies could only provide association and easily flawed by the presence of confounding factors (72). Our result added the evidence that high cholesterol and LDL-C are causal factors of XP. Second, MR analysis could reduce confounding factors and reverse causation bias. Third, a large GWAS dataset provides adequate statistical power for the analysis. However, our study still has limitations. We acquired GWAS data for XP from FinnGen Data Freeze 9. This is the largest dataset containing results for the XP phenotype at present. However, in this dataset, only 228 cases of XP are reported, with 344,684 controls. The small number of cases may limit the statistical power of MR. In addition, MR analysis results can be violated by pleiotropy. In our study, the weighted median and MR-Egger methods provide a consistent direction as IVW, but the influence of horizontal pleiotropy still cannot be excluded. In addition, most of the GWAS studies are mainly from cohorts of European ancestry. It still requires further validation if the conclusion can be generalized to other populations.

## 5 Conclusions

Increased observational studies demonstrated that increased plasma cholesterol was a risk factor for XP. Our MR study further provided evidence for a causal link between plasma cholesterol, LDL-C, and XP, supporting the use of cholesterol-lowering drugs in treating XP. In addition, by analyzing the plasma proteins, we provided evidence that genetically predicted levels of 12 plasma proteins were associated with XP, highlighting the role of cell adhesion and inflammation in the development of XP.

## Data availability statement

The original contributions presented in the study are included in the article/**Supplementary Material**. Further inquiries can be directed to the corresponding authors.

## Author contributions

WH: Conceptualization, Data curation, Formal analysis, Methodology, Writing – original draft. YL: Investigation, Methodology, Supervision, Validation, Writing – review & editing. CL: Conceptualization, Supervision, Validation, Writing – review & editing. HL: Conceptualization, Data curation, Formal analysis, Funding acquisition, Writing – review & editing.

## Supplementary material

Supplementary Table 1 The datasets used in this study.This table describes the sources of the datasets used in this study. N Cases, number of cases; N Controls, numbers of controls; DOI, digital object identifier.

Supplementary Table 2 MR results of plasma lipids to XP. This table is the MR results of total cholesterol, LDL-C, HDL-C, non-HDL-C, and TG to XP, corresponding to **Figure 1**. Method, methods used in MR analysis; N SNP, number of valid SNPs used in MR analysis; Beta, the effect estimated using the genetic variants; SE, standard deviation of beta; OR, Odds ratio with 95% confidence interval; Egger Intercept, the intercept term using MR-Egger method for assessing the pleiotropic effects; Egger Intercept P value, P value of MR-Egger method; Q value, the result of Cochran’s Q test for evaluating the heterogeneity; Q p-value: P value of Cochran’s Q test.

Supplementary Table 3 MR results of metabolic traits to XP. This table is the MR results of BMI, fasting glucose, type 2 diabetes, systolic blood pressure, and diastolic blood pressure to XP, corresponding to **Figure 2**. BMI, body mass index; Method, methods used in MR analysis; N SNP, number of valid SNPs used in MR analysis; Beta, the effect estimated using the genetic variants; SE, standard deviation of beta; OR, Odds ratio with 95% confidence interval; Egger Intercept, the intercept term using MR-Egger method for assessing the pleiotropic effects; Egger Intercept P value, P value of MR-Egger method; Q value, the result of Cochran’s Q test for evaluating the heterogeneity; Q p value: P value of Cochran’s Q test.

Supplementary Table 4 MR results of circulating proteins to XP. The protein quantitative trait loci (pQTLs) are used as genetic IVs in the MR study, and we integrate this plasma proteome with the XP GWAS, correspondent to **Figure 3**. Method, methods used in MR analysis; N SNP, number of valid SNPs used in MR analysis; Beta, the effect estimated using the genetic variants; SE, standard deviation of beta; P-value (FDR adjusted), false discovery rate adjusted P value for multiple testing correction; Egger Intercept, the intercept term using MR-Egger method for assessing the pleiotropic effects; Egger Intercept P value, P value of MR-Egger method; SNP r2 exposure, estimated variance explained in exposure; SNP r2 outcome, estimated variance explained in outcome; Correct causal direction, whether the assumption that exposure causes outcome is valid from MR Steiger test; Steiger P-value, P value of Steiger test; OR, Odds ratio; OR lci95, lower 95% confidence interval of OR; OR uci95, upper 95% confidence interval of OR; Q value, the result of Cochran’s Q test for assessing the heterogeneity; Q p value: P value of Cochran’s Q test.

Supplementary Table 5 MR results of circulating cytokines to XP. The GWAS data of circulating cytokines from Young Finns Study were used as exposure in MR analysis, corresponding to **Figure 4**. Method, methods used in MR analysis; N SNP, number of valid SNPs used in MR analysis; Beta, the effect estimated using the genetic variants; SE, standard deviation of beta; P-value (FDR adjusted), false discovery rate adjusted P value for multiple testing correction; Egger Intercept, the intercept term using MR-Egger method for assessing the pleiotropic effects; Egger Intercept P value, P value of MR-Egger method; SNP r2 exposure, estimated variance explained in exposure; SNP r2 outcome, estimated variance explained in outcome; Correct causal direction, whether the assumption that exposure causes outcome is valid from MR Steiger test; Steiger P-value, P value of Steiger test; OR, Odds ratio; OR lci95, lower 95% confidence interval of OR; OR uci95, upper 95% confidence interval of OR; Q value, the result of Cochran’s Q test for assessing the heterogeneity; Q p value: P value of Cochran’s Q test.

Supplementary Table 6 MR results of circulating inflammatory proteins to XP. The GWAS data of circulating inflammatory proteins from SCALLOP Consortium were used as exposure in MR analysis, corresponding to **Figure 4**. Method, methods used in MR analysis; N SNP, number of valid SNPs used in MR analysis; Beta, the effect estimated using the genetic variants; SE, standard deviation of beta; P-value (FDR adjusted), false discovery rate adjusted P value for multiple testing correction; Egger Intercept, the intercept term using MR-Egger method for assessing the pleiotropic effects; Egger Intercept P value, P value of MR-Egger method; SNP r2 exposure, estimated variance explained in exposure; SNP r2 outcome, estimated variance explained in outcome; Correct causal direction, whether the assumption that exposure causes outcome is valid from MR Steiger test; Steiger P-value, P value of Steiger test; OR, Odds ratio; OR lci95, lower 95% confidence interval of OR; OR uci95, upper 95% confidence interval of OR; Q value, the result of Cochran’s Q test for assessing the heterogeneity; Q p value: P value of Cochran’s Q test.

Supplementary Table 7 SNPs used for MR analysis. This table lists all SNPs used as IVs for MR analysis in this study, corresponding to **Figures 1**, **2**. CHR, chromosome; POS, position on chromosome; Other allele, reference allele; Beta, the effect estimated using the genetic variants; SE, standard deviation of beta; F, F-statistic for average strength.
